# praja2 regulates KSR1 stability and mitogenic signaling

**DOI:** 10.1038/cddis.2016.109

**Published:** 2016-05-19

**Authors:** L Rinaldi, R Delle Donne, M Sepe, M Porpora, C Garbi, F Chiuso, A Gallo, S Parisi, L Russo, V Bachmann, R G Huber, E Stefan, T Russo, A Feliciello

**Affiliations:** 1 Dipartimento di Medicina Molecolare e Biotecnologie Mediche, IEOS-CNR, CEINGE University Federico II, Naples 80131, Italy; 2 Institute of Biochemistry and Center for Molecular Biosciences, University of Innsbruck, Innsbruck, Austria; 3 Bioinformatics Institute (BII), Agency for Science Technology and Research (A*STAR), Singapore 138671, Singapore

## Abstract

The kinase suppressor of Ras 1 (KSR1) has a fundamental role in mitogenic signaling by scaffolding components of the Ras/MAP kinase pathway. In response to Ras activation, KSR1 assembles a tripartite kinase complex that optimally transfers signals generated at the cell membrane to activate ERK. We describe a novel mechanism of ERK attenuation based on ubiquitin-dependent proteolysis of KSR1. Stimulation of membrane receptors by hormones or growth factors induced KSR1 polyubiquitination, which paralleled a decline of ERK1/2 signaling. We identified praja2 as the E3 ligase that ubiquitylates KSR1. We showed that praja2-dependent regulation of KSR1 is involved in the growth of cancer cells and in the maintenance of undifferentiated pluripotent state in mouse embryonic stem cells. The dynamic interplay between the ubiquitin system and the kinase scaffold of the Ras pathway shapes the activation profile of the mitogenic cascade. By controlling KSR1 levels, praja2 directly affects compartmentalized ERK activities, impacting on physiological events required for cell proliferation and maintenance of embryonic stem cell pluripotency.

The Ras-Raf-ERK protein kinase cascade constitutes a central signaling mechanism that controls important cell functions, such as differentiation, metabolism and cell growth. Activation of Ras by growth factors or G protein-coupled receptor (GPCR) ligands promotes a kinase suppressor of Ras 1 (KSR1)-mediated formation of a tripartite kinase complex, which compartmentalizes Raf, MEK and ERK.^1, 2, 3^ By juxtaposing upstream and downstream signaling kinases, KSR1 optimally couples stimulation of membrane receptors to the propagation of the signals to a variety of ERK substrates controlling multiple biological activities, such as cell proliferation, metabolism and synaptic activity.^4, 5, 6, 7, 8, 9^ Dysregulation or mutations in the genes encoding components of this transduction pathway are frequently found in several human cancers.^10, 11, 12^ Interfering with KSR1 function reduces Ras signaling and cancer cell growth.^13, 14, 15, 16, 17^ Distinct attenuation mechanisms of signaling cascade have been identified.^18, 19, 20^ As for mitogenic pathway, a negative loop between ERK1/2 and KSR1 ensures an efficient and tightly controlled cycle of activation/de-activation process that limits unrestrained and widespread activation of mitogenic signaling. Phosphorylation of KSR1 and B-Raf by locally activated ERK1 dissociates the KSR1 multi-kinase complex, turning-off the ERK cascade.^15, 20^ The mitogenic cascade could also be firmly regulated through phosphorylation of Raf and KSR1 by cAMP-dependent protein kinase (PKA).^21^ The bi-directional regulation of KSR1 and ERK cascade, and the integration of the Ras pathway with signals carried out by the GPCR•cAMP signaling axis control the rate, magnitude and persistence of the downstream mitogenic pathway.

The ubiquitin–proteasome system (UPS) emerged as an important posttranslational mechanism that controls cell growth, differentiation, metabolism and survival. The UPS couples ubiquitylation of a target protein to its proteolytic cleavage, eliminating unneeded or damaged proteins and contributing to essential aspects of cell signaling and homeostasis.^22^ The process involves the sequential action of ubiquitin-activating enzymes (E1), ubiquitin-conjugating enzymes (E2) and ubiquitin ligases (E3), where each enzyme transfers ubiquitin molecules from one enzyme to the next and eventually to the target protein. praja2 belongs to a growing family of widely expressed mammalian RING-H2 proteins with intrinsic E3 ubiquitin-ligase activity.^23, 24, 25, 26^ During GPCR•cAMP stimulation, praja2 ubiquitylates and degrades the inhibitory (R) subunits of PKA, sustaining downstream signals carried out by cAMP.^27^ In proliferating cells, praja2 promotes ubiquitin-dependent proteolysis of MOB1, a core component the tumor-suppressor Hippo cascade. Degradation of MOB1 through the UPS attenuates the Hippo cascade and sustains tumor growth.^28^ A role of praja2•UPS in neuronal differentiation and glucose homeostasis has also been recently described.^29, 30^ However, the impact of praja2 in the control of ERK signaling was unknown.

Here we report that the KSR1 abundance is controlled by specific components of the ubiquitin pathway. We identified praja2 as the E3 ligase that ubiquitylates KSR1 in response to growth factor or cAMP stimulation. Ubiquitination of KSR1 eventually attenuates the ERK1/2 cascade. By modulating KSR1•ERK signaling, praja2 profoundly impacts on essential aspects of embryonic stem cell (ESC) differentiation.

## Results

### Identification of KSR1 as novel praja2 interactor

**Figure 1:**
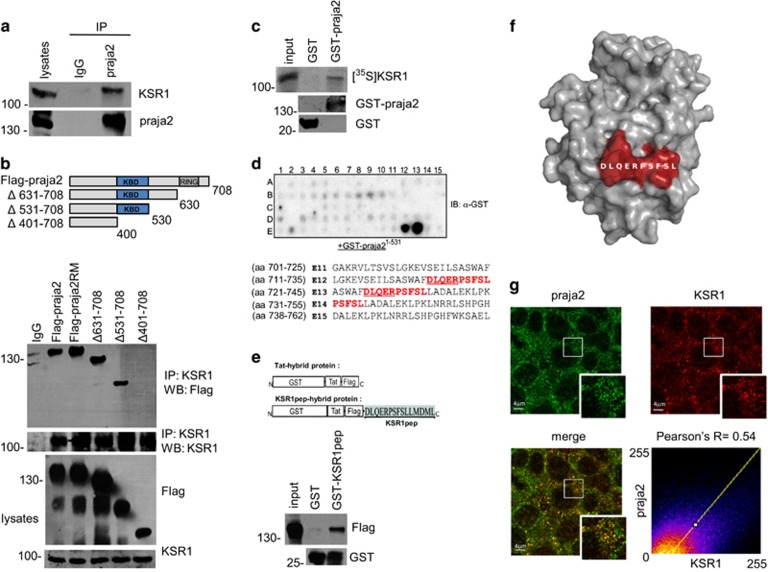
praja2 interacts with KSR1. (**a**) Isolation of endogenous KSR1 and praja2 complex from lysates (2 mg) of HEK293 cells. (**b**) Schematic representation of the praja2 constructs used (upper diagram). HEK293 cells were transiently transfected with flag-praja2 (either wild-type, ring mutant (RM) or deletion mutants). Cells were treated for 12 h with MG132 (10 *μ*M) before harvesting. Twenty-four hours following transfection, cells were harvested and lysed. Lysates were subjected to immunoprecipitation with anti-KSR1 antibody. Precipitates were immunoblotted with anti-KSR1 and anti-flag antibodies (lower panels). (**c**) *In vitro* translated, [^35^S]-labeled KSR1 was subjected to pull-down assays with purified GST or GST–praja2 fusion. (**d**) Spotted peptides (25 mers, 15mer overlap) of the human KSR1 sequence were overlaid with recombinant GST-praja2 (1–531) followed by immunoblotting with anti-GST antibody. The sequences (in red) refer to the praja2-binding domain of KSR1. The amino acids methionine and cysteine have been substituted with alanine or serine, respectively. (**e**) Schematic representation of the peptides used for pull-down experiments (upper panel). Lysates from flag-praja2-transfected cells were subjected to pull-down experiment with GST or GST-KSR1pep carrying the praja2-binding domain fused to GST (lower panels). (**f**) Predicted structure of KSR1 kinase domain modeled on KSR2 template PDB 2Y4I. The position of the potential praja2-binding domain is indicated. (**g**) HEK293 cells were subjected to double immunostaining with monoclonal anti-KSR1 and polyclonal anti-praja2 antibodies. Images were collected and analyzed by confocal microscopy. Pearson's coefficients between praja2 and KSR1

By controlling the stability of protein kinases (PKA and Lats/Mob1), praja2 integrates signals carried out by two evolutionary conserved transduction cascades, having a major role in cell proliferation and tumor growth.^28^ Large-scale proteomic analyses revealed that praja2 is a component of a macromolecular complex that includes the ERK scaffold KSR1.^31^ Based on this finding, we tested whether praja2 interacts with and regulates the stability KSR1. First, we demonstrated the interaction *in vivo* by isolating an endogenous praja2/KSR1 complex from cell lysates (Figure 1a). Similarly, exogenous flag-praja2 and co-expressed myc-KSR1 formed a stable complex in cell lysates (Figure 1b). The core domain of praja2 (residues 401–530) was required for KSR1 binding (Figure 1b). Next we confirmed a direct interaction between KSR1 and praja2 *in vitro*. A fusion protein carrying full-length praja2 fused to the C-terminus of glutathione *S*-transferase polypeptide (GST) co-precipitated in *in vitro*-translated, [^35^S]-labeled KSR1 (Figure 1c). To map the praja2 interacting motif, overlapping 25-mer peptides derived from human KSR1 sequence were spotted onto a membrane and overlaid with purified GST-praja2^1–531^ fusion protein, as previously described.^32^ We identified one possible binding site for praja2 at the C-terminus of KSR1 with the core region DLQERPSFSL (Figure 1d). As expected, a recombinant protein carrying the core peptide of KSR1 fused at the C-terminus of the GST polypeptide was sufficient to bind praja2 *in vitro* (Figure 1e). The potential binding site is, indeed, located at the surface of the predicted KSR1 structure, and thus it should be easily accessible for praja2 interaction (Figure 1f). *In* *situ* immunofluorescence analysis revealed a partial co-localization between endogenous praja2 and KSR1. Overlapping signals could be detected at the perinuclear region and within the cytoplasm (Figure 1g), with a mean Pearson's coefficient of ~0.5. Altogether, these data support the existence of a praja2/KSR1 complex in living cells.

### praja2 ubiquitylates KSR1

**Figure 2:**
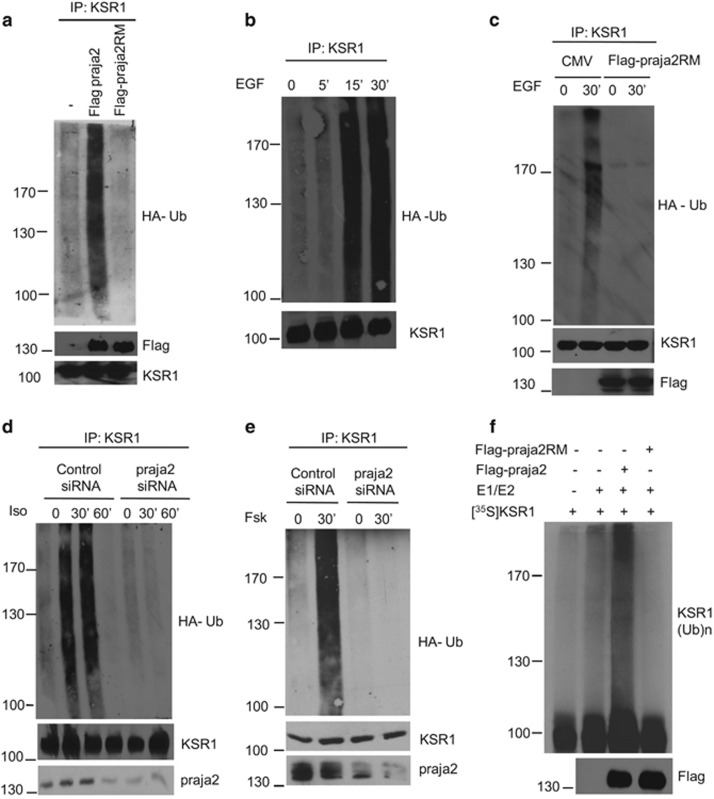
praja2 ubiquitylates KSR1. (**a**) HEK293 cells were transfected with HA-ubiquitin and flag-praja2 or flag-praja2RM. Twenty-four hours after transfection, cells were treated with MG132 (20 *μ*M) for 8 h. Lysates were subjected to immunoprecipitations with anti-KSR1 and immunoblotted with anti-HA and anti-flag antibodies. (**b**) Cells were transfected with HA-ubiquitin, serum-deprived overnight and stimulated with EGF (100 ng/ml) for the indicated time points. Lysates were subjected to immunoprecipitations with anti-KSR1 and immunoblotted with anti-HA and anti-KSR1 antibodies. (**c**) Cells were transfected with HA-ubiquitin and flag-praja2 or flag-praja2RM and processed as in panel (**b**). (**d**) HEK293 cells were transfected with HA-ubiquitin and control (siRNAc) or SMARTpool siRNApraja2. Twenty-four hours after transfection, cells were either left untreated or stimulated with isoproterenol in the presence of MG132. Lysates were subjected to immunoprecipitations with anti-KSR1 and immunoblotted with anti-HA and anti-KSR1 antibodies. (**e**) Same as in panel (**d**), with the exception that the cells were stimulated with Fsk (40 *μ*M, 30 min). (**f**) *In vitro*-translated, ^35^S-labeled KSR1 was incubated with anti-flag precipitates (flag-praja2 or flag-praja2RM) isolated from growing cells and his_6_-tagged ubiquitin, in the presence of E1 and UbcH5c (E2). The reaction mix was denatured, size-fractionated on SDS-PAGE and analyzed by autoradiography. A fraction of the reaction mixture was immunoblotted with anti-flag antibody (lower panel)

As praja2 is an E3 ubiquitin ligase, we asked whether praja2 ubiquitylates KSR1. As predicted, overexpression of praja2 induced accumulation of polyubiquitinated forms of co-transfected KSR1 (Figure 2a). In contrast, expression of a ligase inactive mutant of praja2 (praja2RM) reduced ubiquinated KSR1 levels below the control value. Stimulation with epidermal growth factor (EGF) induced a rapid and efficient accumulation of polyubiquitinated KSR1 (Figure 2b). Stimulation by EGF of KSR1 ubiquitination was prevented by praja2RM expression (Figure 2c). Importantly, stimulation of *β*2-adrenergic receptor with isoproterenol promoted KSR1 ubiquitination by praja2 (Figure 2d). PKA is a relevant effector of GPCR signaling. Therefore, we tested whether general cAMP mobilization and PKA activation *per se* contribute to KSR1 ubiquitination. As shown in Figure 2e, treatment with the cAMP-elevating agent Forskolin (Fsk) rapidly induced KSR1 polyubiquitination. Genetic knockdown (KD) of praja2 prevented Fsk-induced KSR1 ubiquitination. *In vitro* ubiquitination assays demonstrated that praja2, but not its inactive mutant, directly ubiquitylates KSR1 (Figure 2f).

**Figure 3:**
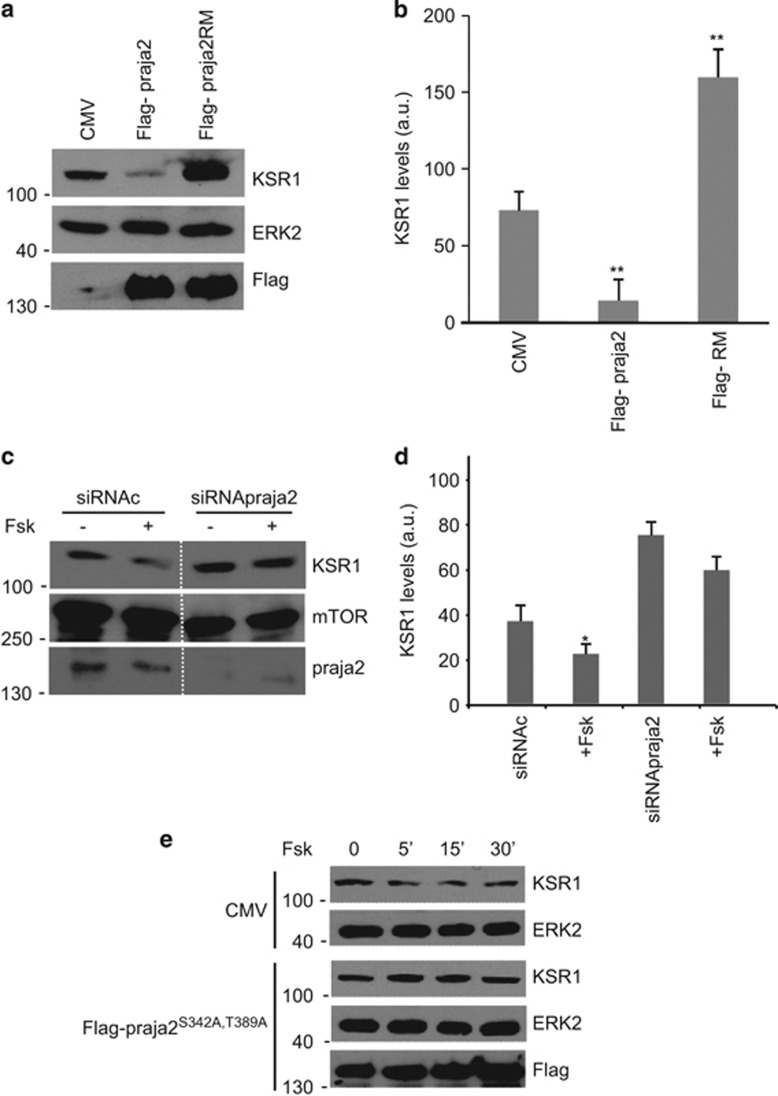
praja2 decreases KSR1 levels. (**a**) Immunoblot of lysates from cells transiently transfected with Flag-praja2 vector (either wild type or RING mutant). (**b**) Quantitative analysis (mean±S.E.M) of three independent experiments shown in panel (**a**) **P<0.01. (**c**) Cells were transfected with control (siRNAc) or SMARTpool siRNApraja2. Twenty-four hours after transfection, cells were either left untreated or stimulated with Fsk (40 *μ*M, 30 min). To prevent effects of Fsk on protein synthesis, cells were pretreated with cycloheximide. (**d**) Quantitative analysis of three independent experiments shown in panel (**e**). *P<0.05 *versus* control (siRNAc), untreated cells. (**e**) Immunoblot of lysates from cells transfected with flag-praja2 vector (either wild type or praja2^S342A,T389A^). Twenty-four hours after transfection, cells were either left untreated or stimulated with Fsk (40 *μ*M) and harvested at the indicated time points

These findings demonstrate that KSR1 is targeted by the ubiquitin pathway in response to stimulation of receptor tyrosine kinase (RTK) or GPCR. This implies that praja2 will reduce KSR1 levels. We tested this hypothesis by measuring KSR1 in cells expressing praja2 (either wild type or RING mutant). As shown in Figures 3a and b, praja2 significantly lowered, whereas praja2RM increased, the levels of endogenous KSR1, relative to the control. Pretreating the cells with the proteasome inhibitor MG132 partly restored KSR1 levels, even in the presence of overexpressed praja2 (Supplementary Figure S1), supporting a role of the proteasome in controlling KSR1 stability.

Our ubiquitination experiments suggest that elevated cAMP levels elicited by GPCR activation or Fsk treatment induced KSR1 ubiquitination by praja2. We therefore asked whether cAMP accumulation was sufficient to reduce KSR1 levels. As shown in Figures 3c and d, the levels of KSR1 decreased following Fsk treatment. The effects of Fsk on KSR1 were reversed by praja2 depletion (Figures 3c and d) or by expressing a praja2 mutant (Flag-praja2^S342A,T389A^) that cannot be phosphorylated and activated by PKA (Figure 3e).^27^

### praja2 attenuates KSR1-dependent ERK1/2 phosphorylation

**Figure 4:**
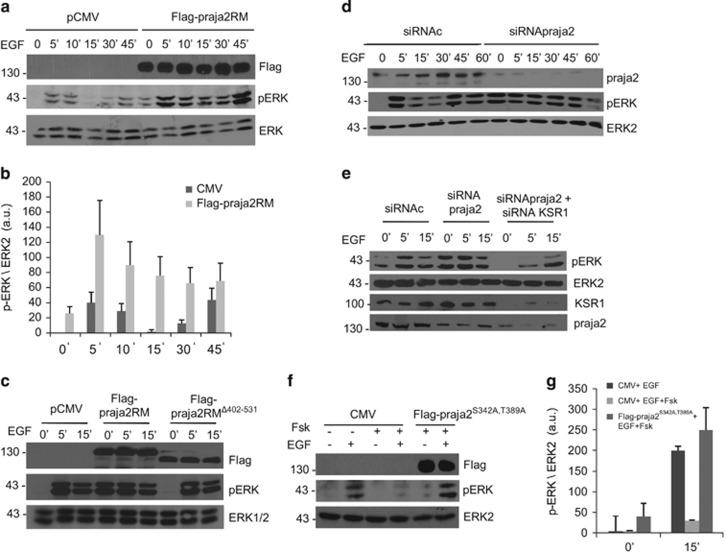
praja2 regulates ERK1/2 signaling. (**a**) HEK293 cells were transiently transfected with control (CMV) or Flag-praja2RM, serum deprived and then treated with EGF (100 ng/ml). Lysates were immunoblotted with the indicated antibodies. (**b**) Quantitative analysis of the experiments shown in panel (**a**). (**c**) HEK293 were transfected with Flag praja2RM mutant or Flag-praja2 RM^Δ402–531^ and treated with EGF for 5 and 15 min. Lysates were immunoblotted with the indicated antibodies. (**d**) Cells were transfected with control (siRNAc) or SMARTpool siRNApraja2, serum deprived and stimulated with EGF. Lysates were immunoblotted with the indicated antibodies. (**e**) Cells were transfected with control (siRNAc), SMARTpool siRNApraja2 or SMARTpool siRNApraja2 and siRNA-KSR1 and stimulated with EGF. Lysates were immunoblotted with the indicated antibodies. (**f**) Cells were transfected with pCMV vector or Flag-praja2^S342A,T389A^. Twenty-four hours after transfection, cells were stimulated with Fsk (40 *μ*M) and harvested at the indicated time points. (**g**) Quantitative analysis of the experiments shown in panel (**e**)

The data above indicate that praja2 regulates the KSR1 stability. Given the role of KSR1 in ERK1/2 signaling, we investigated whether praja2 modulates ERK activity. To this end, we monitored ERK1/2 phosphorylation at its active site (Thr202/Tyr204) in cells stimulated with EGF. As shown in Figures 4a and b, we observed that EGF treatment induced a time-dependent phosphorylation of ERK1/2. Expression of praja2RM increased both basal and EGF-induced ERK phosphorylation by several fold over control values. The high levels of basal ERK phosphorylation in praja2RM-transfected cells support the role of endogenous praja2 in attenuating ERK activity in serum-deprived cells. The same experiments were replicated in human osteosarcoma cells (U2OS) (Supplementary Figure S2A). The effects of praja2RM on ERK1/2 phosphorylation was inhibited by pretreating the cells with the MEK inhibitor U0126 (Supplementary Figure S2B). The interaction to KSR1 was required for praja2RM action. Thus a praja2RM lacking the KSR-binding domain had no effects on ERK1/2 phosphorylation profile (Figure 4c). Similarly, genetic KD of endogenous praja2 also sustained ERK1/2 phosphorylation, compared with controls (Figure 4d and Supplementary Figure S3).

ERK phosphorylation in praja2 siRNA-transfected cells was downregulated by concomitant KSR1 depletion (Figure 4e), suggesting that KSR1 is a relevant target of praja2 in shaping ERK signaling. Next we tested whether praja2 regulates ERK phosphorylation in the course of GPCR stimulation.

cAMP accumulation within cells inhibits the ERK1/2 cascade by different mechanisms.^33, 34, 35^ The data above indicate that cAMP promotes ubiquitination of KSR1 through praja2. This suggested that cAMP may inhibit ERK phosphorylation by promoting KSR1 ubiquitination. We tested this hypothesis by monitoring ERK phosphorylation in cells exposed to EGF in the presence or absence of Fsk. Figures 4f and g show that cAMP inhibits ERK phosphorylation induced by EGF, as described previously.^33, 34, 35^ cAMP inhibition of ERK signaling was abrogated by the praja2^S342A,T389A^ mutant (Figures 4f and g), supporting a key role of the ligase in mediating cAMP effects on ERK cascade.

**Figure 5:**
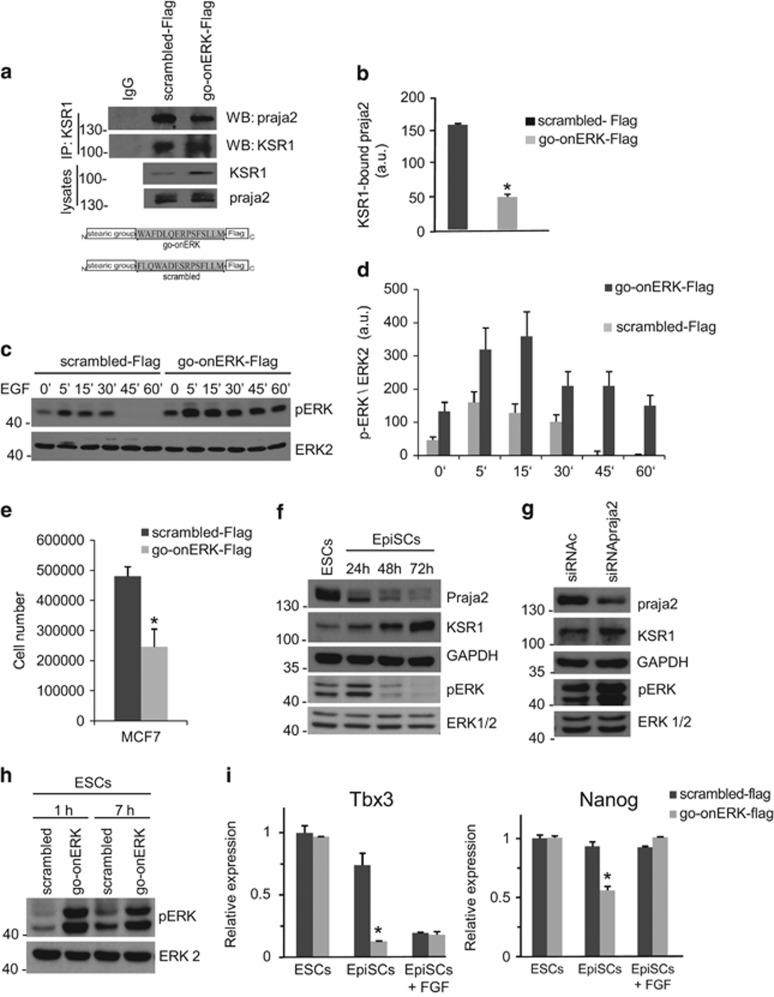
Interfering with praja2-KSR1 complex enhances ERK signaling in ES and cancer cells. (**a**) Cells were pretreated for 8 h with the following synthetic peptides (1 *μ*M): scrambled-Flag and KSR1pep-Flag, renamed as go-onERK-Flag, given its role in ERK signaling (see below). Lysates were immunoprecipitated for KSR1. The precipitates were immunoblotted for praja2 and KSR1 (upper panel). Schematic representation of the scrambled and go-onERK peptides (lower panel). (**b**) Quantitative analysis of the experiments shown in panel (**a**). The data represent a mean of two independent experiments. (**c**) Cells pretreated (7 h) with the synthetic peptides (1 *μ*M) (scrambled-Flag and go-onERK-Flag) were left untreated or stimulated with EGF. Lysates were immunoblotted for phosphoERK and ERK. (**d**) Quantitative analysis of the experiments shown in panel (**c**). (**e**) MCF-7 cells were treated for 48 h with the scrambled-Flag peptide or with go-onERK-Flag peptide, harvested and counted. A mean of three independent experiments±S.E.M. is shown. (**f**) E14Tg2a mouse ESCs were grown on feeder-free, gelatin-coated plates as described^49^ and were induced to differentiate into EpiSCs in fibronectin-coated dishes at a density of 2.5 × 10^5^cells/cm^2^ in the presence of 20 ng/ml Activin A and 12 ng/ml bFGF as described.^50^ Lysates from cells were immunoblotted with the indicated antibodies. (**g**) Undifferentiated ESCs were transfected with control (siRNAc) or SMARTpool siRNApraja2. Lysates were immunoblotted with the indicated antibodies. (**h**) Cells treated (1 and 7 h) with the synthetic peptides (scrambled-Flag and go-onERK-Flag-Flag) or with 12 ng/ml bFGF. Lysates were immunoblotted for the indicated antibodies. (**i**) ESCs were grown in undifferentiated conditions in the presence of LIF and serum (ESCs) or in EpiSC medium (containing Activin) for 48 h with bFGF (EpiSCs+bFGF) or without it (EpiSCs), in the presence of scrambled-Flag or go-onERK-Flag. Q-PCR analysis of Tbx3 and Nanog mRNAs, relative to GAPDH mRNA. The results are expressed as mean±S.E.M of three independent experiments (*P<0.05)

We next showed that binding to KSR1 was required for praja2 inhibition of ERK1/2 signaling. Cells were pretreated for 6 h with a synthetic peptide spanning the praja2-binding motif of KSR1 (go-onERK-Flag) or with a scrambled peptide (scrambled-Flag). The peptides were linked to a stearate group to facilitate the cellular uptake (Figure 5a, lower panel). As shown in Supplementary Figure S4, both peptides efficiently accumulated within the treated cells. As expected, treatment with go-onERK-Flag decreased praja2 binding to KSR1 (Figure 5a, upper panel and Figure 5b). The cells were then stimulated with EGF and ERK1/2 phosphorylation was monitored over a time-point curve. Pretreatment with go-onERK-Flag enhanced both basal and EGF-induced ERK phosphorylation compared with controls (Figures 5c and d). The effects of KSR1go-onERK-flag on ERK phosphorylation were replicated in other cell lines, including LNCAP and U-87 MG (Supplementary Figure S5). We also monitored the effects of the peptide treatment on the growth of human cancer cells. As shown in Figure 5e, KSR1go-onERK-flag inhibited the growth of MCF-7 by about twofolds, compared with controls.

### praja2 regulates ERKs in ESCs

In addition to mitogenic signaling, ERKs are crucially involved in cell differentiation. Stimulation of the ERK1/2 cascade by basic fibroblast growth factor (bFGF) governs mouse ESC fate, regulating the transition between naive pluripotency and epiblast-like state committed to differentiation (epiblast-like stem cells (EpiSCs)).^36, 37, 38, 39^ We observed that both praja2 and KSR1 are expressed in both ESCs and EpiSCs (Figure 5f). The levels of praja2 and KSR1 are inversely correlated during the transition from ESCs to EpiSCs. The boost of ERK activation soon after the transition induction is concomitant with the decrease of praja2 and the accumulation of KSR1 (Figure 5g) and is in agreement with the function of Erk1/2 in triggering transition of pluripotent ESCs from ground to a primed state.^36^ It is worth noting that P-ERK level declines soon after the boost of activation, likely due to the activation of phosphatase Dusp6,^40^ whereas KSR1 continues to accumulate. This suggests that KSR accumulation is also involved in further functions, different from that leading to transient ERK activation. Suppression of praja2 by RNAi in ESCs induced the accumulation of KSR1 and was accompanied by a robust phosphorylation of ERKs (Figure 5g), thus indicating that downregulation of KSR1 by praja2 could contribute to maintain appropriate levels of ERK phosphorylation in ESCs. ERK1/2 phosphorylation was also observed in ESCs exposed to the go-onERK-Flag peptide (Figure 5h) and was comparable to that provoked by the treatment with bFGF.^36^

To explore the role of praja2 in ERK-dependent ESCs differentiation, we exposed ESCs to go-onERK-Flag peptide in a medium without LIF and serum and with activin, which favors the exit from naive pluripotency.^41^ We first assessed whether ERK1/2 induction by go-onERK-Flag peptide activates the downstream pathways. It is well known that ERK activation during the transition from ESCs to EpiSCs downregulates the transcription of Tbx3, a known downstream target of ERK signaling.^39^ As shown in Figure 5i, exposure of ESCs to bFGF or to go-onERK-Flag peptide resulted in the downregulation of Tbx3. Considering that Nanog gene is, in part, under the control of Tbx3, we asked whether go-onERK-Flag peptide affected Nanog gene expression. Figure 5i shows that Nanog mRNA was decreased upon exposure of the cells to go-onERK-Flag peptide. Therefore, the induction of ERKs by go-onERK-Flag peptide appeared to have the expected physiological downstream effect. However, go-onERK-Flag treatment alone failed to induce the differentiation of ESCs, thus confirming that, in addition to ERK activation, other events, such as the induction of GSK3*β*, are necessary to sustain ESC differentiation into EpiSCs.

## Discussion

Here we report for the first time the identification of KSR1 as a target of the UPS. We identified praja2 as the E3 ligase responsible for KSR1 ubiquitination. We found that, in the course of hormone stimulation by RTKs and GPCRs ligands, KSR1 undergoes rapid polyubiquitination and proteolysis, which eventually leads to attenuation of ERK-mediated signal transmission.

Signal propagation originating at cell membranes is efficiently transmitted to compartmentalized downstream effectors. To ensure specificity, it is also necessary to restrict signaling temporally by desensitizing the transmitter of the input signal. The intricate interplay between ON and OFF states of signaling pathways gives rise to signaling waves that rapidly propagate throughout the cell, eliciting biological responses that strictly depend on the intensity and frequency of the oscillatory circuits. A variety of attenuation mechanisms have been identified.^18, 19, 20^ In the case of the ERK pathway, a negative loop between ERK and KSR1 ensures a cycle of activation/de-activation process that limits uncontrolled mitogenic signaling. Phosphorylation of KSR1 and B-Raf by locally activated ERK1 dissociates the KSR1 multi-kinase complex, turning-off the ERK cascade.^15, 20^ Phosphorylation of Raf and KSR1 by PKA also modulates ERK activity.^21^ The bi-directional regulation of KSR1 and ERK cascade and the integration of the Ras pathway with signals carried out by cAMP ultimately control the rate, magnitude and persistence of the downstream mitogenic pathway.

Previous findings indicated that KSR1 protein stability could be regulated at the posttranslational level.^8, 42^ However, the mechanisms underlying the control of KSR1 stability were unknown. Our work adds a novel twist in the ERK cascade, identifying KSR1 as a target of the UPS. Ubiquitination of KSR1 is rapidly induced by cAMP•PKA pathway. Ubiquitinated KSR1 eventually undergoes proteolysis through the proteasome. This parallels downregulation of the ERK cascade. Interfering with praja2 expression/activity or expressing a praja2 mutant carrying mutations within the PKA phosphorylation sites negatively impacted on KSR1 ubiquitination, enhancing ERK1/2 activation and prolonging the wave of downstream signaling. Moreover, a peptide targeting the praja2-binding interface on KSR1 increased both basal and ligand-induced ERK1/2 phosphorylation, supporting the role of praja2 in negatively controlling the KSR1 signaling. praja2 acts also in response to EGF stimulation in promoting KSR1 ubiquitination and ERK attenuation, pointing to a more general role of praja2 in controlling the strength and duration of KSR1-orchestrated ERK cascade. The biological relevance of this mechanism was supported by the finding that interfering with praja2/KSR1 interaction significantly affected the growth rate of human cancer cells. In this context, inhibition of tumor cells growth by the praja2/KSR1 displacing peptide may provide a new therapeutic strategy to selectively interfere with the growth and development of malignant lesions.

The functional relevance of KSR1 regulation by praja2 also emerged in mouse ESCs. Tight control of ERK1/2 activation is known to be crucial to maintain ESCs in the pluripotent ground state and to drive differentiation of these cells into the epiblast-like phenotype.^36, 39, 43^ The fundamental role of this control is supported by the observation that the pluripotent ground state can be sustained, in the absence of LIF and serum, by inhibitors of ERKs and GSK3*β*.^37^ We show that praja2 regulates KSR1 abundance in ESCs and, in turn, supports the ERK activation that is crucial for ESC differentiation. This observation sheds new light of the molecular events involved in the transition from preimplantation into postimplantation phenotype of epiblast cells and suggests to further explore the regulation of this machinery in the maintenance of pluripotency, in the differentiation of ESC and in the reprogramming.

In conclusion, our findings highlight the importance of praja2•UPS in the control of growth factor signaling, pointing to KSR1 as a relevant target of praja2. Identification of a core motif within the KSR1 structure that mediates praja2 binding provides a potential strategy to interfere selectively with Ras•KSR1•ERK signaling in living cells. Exploring further the mechanism(s) regulating ubiquitination of KSR1 in course of hormone and growth factor stimulation and identifying additional UPS targets in the ERK cascade will provide essential tools to dissect and manipulate these important signaling pathways in human diseases.

## Materials and Methods

### Cell lines

Human embryonic kidney cell line (HEK293), glioblastoma cells (U87-MG) and osteosarcoma cells (U2OS) were cultured in Dulbecco's modified Eagle's medium containing 10% fetal bovine serum in an atmosphere of 5% CO_2_. To favor EpiSC differentiation, 10^6^ cells per 10 cm^2^ were plated on TC dishes pretreated with Fibronectin (Sigma, Saint Louis, MO, USA) in the following medium: 1 volume of DMEM/F12 combined with 1 volume of Neurobasal medium, supplemented with 0.5% N2 supplement, 1% B27 supplement, 1% KO serum replacement, 2 mM glutamine (Invitrogen, Carlsbad, MO, USA), 20 ng/ml Activin A (R&D Systems, Minneapolis, MN, USA) with or without 12 ng/ml of bFGF (Preprotech). Within 2 days in these conditions, the cells undergo morphological transformation (including flattening, diminished cell–cell interactions and formation of cellular protrusions) and express epiblast markers.^41^

### Plasmids and transfection

Vectors encoding for flag-praja2 (either wild type or mutants) and GST-praja2 were purchased from (Genecopeia, Rockville, MD, USA); HA-tagged ubiquitin was previously described;^44^ KSR1 vectors were kindly provided by Dr. Richard Kolesnick and Dr. Andrey Shaw. SMART pool siRNAs targeting distinct segments of coding regions of praja2 and KSR1 were purchased from (Thermo Scientific, Waltham, MA, USA) and Sigma. siRNAs were transiently transfected using Lipofectamine 2000 (Invitrogen) at a final concentration of 100 pmol/ml of culture medium. For siRNA experiments, similar data were obtained using a mixture or four or two independent siRNAs. Transfection efficiency was monitored by including a GFP vector in the transfection mixture.

Following are the siRNA sequences (Thermo Scientific; LU-006916-00-10) targeting human praja2:

Sequence 1: 5′-GAAGCACCCUAAACCUUGA-3′

Sequence 2: 5′-AGACUGCUCUGGCCCAUUU-3′

Sequence 3: 5′-GCAGGAGGGUAUCAGACAA-3′ and

Sequence 4: 5′-GUUAGAUUCUGUACCAUUA-3′.

### RNA isolation and quantitative real-time PCR (Q-PCR)

Total RNA was extracted by using TRISURE (Bioline, Taunton, MA, USA). The first-strand cDNA was synthesized using the M-MLV RT Kit (New England BioLabs, Ipswich, MA, USA) according to the manufacturer's instructions. Q-PCR was carried out with the QuantStudio 7 Flex Real-Time PCR System instrument and software (Applied Biosystems, Carlsbad, CA, USA) using Power SYBR Green PCR Master mix (Applied Biosystems). The housekeeping GAPDH mRNA was used as an internal standard for normalization. Gene-specific primers used for amplification are listed below.

Tbx3:

5′-CGAAGTCAGGAAGGCGAATG-3′

5′-TGTCCATCAATAAAATATACTTGGCC-3′

Gapdh:

5′-GTATGACTCCACTCACGGCAAA-3′

5′-TTCCCATTCTCGGCCTTG-3′

Nanog:

5′-TCAGAAGGGCTCAGCACCA-3′

5′-GCGTTCACCAGATAGCCCTG-3′

### Antibodies and western blotting analysis

Cells were lysed in a buffer containing 1 mM EDTA, 50 mM Tris-HCl (pH 7.5), 70 mM NaCl and 1% Triton. The following primary antibodies were used: rabbit Praja2 (Bethyl Laboratories, Montgomery, TX, USA), mouse GAPDH (Santa Cruz Biotechnology, Dallas, TX, USA), rabbit KSR1 (Cell Signalling, Danvers, MA, USA), rabbit Phospho ERK (Thr202/Tyr204) (Cell Signalling), rabbit ERK2 (Santa Cruz) and rabbit ERK1 (Santa Cruz). Antibody protein complexes were detected by HRP-conjugated antibodies and ECL (both from Amersham Pharmacia, Piscataway Township, NJ, USA).

### Immunoprecipitation and pull-down assay

Cells were homogenized and subjected to immunoprecipitation and immunoblot analyses as described previously.^29^ GST-fusions were expressed and purified from BL21 (DE3) pLysS cells. GST hybrid proteins (GST and GST-pep) immobilized on glutathione beads were incubated for 3 h with cell lysates from HEK293 cells transiently expressing flag-praja2. For *in vitro*-binding assays, 20 *μ*l of GST and GST-praja2 beads were incubated in *in vitro*-translated, [^35^S]-labeled KSR1 in 200 *μ*l lysis buffer (150 mM NaCl, 50 mM Tris-HCl pH 7.5, 1 mM EDTA, 0.5% triton X-100) in rotation at 4 °C overnight. Pellets were washed four times in lysis buffer supplemented with NaCl (0.4 M final concentration) and eluted in Laemmli buffer. Eluted samples were size-fractionated on SDS-PAGE and immunoblotted or subjected to autoradiography.

### *In vitro* ubiquitination assay

[^35^S]-labeled KSR1 was synthesized *in vitro* using TnT quick coupled transcription/translation system (Promega, Fitchburg, WI, USA) in the presence of 45 *μ*Ci of [^35^S]-labeled methionine. The ubiquitination assay was performed as described previously.^27^

### SPOT synthesis and overlay experiments

Overlapping peptides of human KSR1 were SPOT-synthesized (synthetic peptide arrays on membrane supports) on distinct coordinates of a cellulose membrane (starting from the N-terminus of KSR1 with amino acid 1–25 (coordinate A1), amino acid 11–35 (coordinate A2), etc.). The amino acids methionine and cysteine have been substituted with alanine or serine respectively. Membranes equilibrated in TBST buffer (10 mM Tris, 150 mM NaCl, 0,05% Tween 20, pH 7,4) were overlaid with recombinant GST-praja2 (1–531) (10 *μ*g/ml blocking buffer (TBST supplemented with 5% non-fat dry milk)). Interactions were detected with rabbit anti-GST and secondary horseradish peroxidase antibodies by a procedure identical to immunoblotting.

### Immunofluorescence and confocal analysis

For immunofluorescence study, HEK293 cells were plated on poly-l-lysine coated (10 *μ*g/ml) glass coverslips. Cells were fixed and immunostained with ant-KSR1 (1 : 100) and anti-praja2 (1 : 500) antibodies for the endogenous protein levels, with anti-Flag (1 : 500) antibody for the peptides. Immunofluorescence was visualized using a Zeiss LSM 510 Meta argon/krypton laser scanning confocal microscope (Oberkochen, Germany). Quantification of the immunofluorescent images and correlation (Pearson's) coefficient were calculated by the Image-J software (NIH, Bethesda, MD, USA).

### Homology modeling

The sequence of human KSR1 was retrieved from Uniprot (KSR1_HUMAN, Q8IVT5) and aligned with the sequence of KSR2 (KSR2_HUMAN, Q6VAB6) using Clustal Omega at EMBL-EBI.^45, 46^ The result of this alignment is shown in Supplementary Figure S6. The structure of the kinase domain of KSR2 has been determined by Brennan *et al.*^47^ (PDB 2Y4I). Residues with known structure are indicated in yellow. Subsequently, a homology model based on this structural template was created using MOE2013.08. Ten intermediate models were generated and energy minimization was performed for each model using the Amber force field ff99SB^48^ and implicit solvation. The lowest energy state of the 10 models was subsequently chosen as the final model.

## Abbreviations

KSR: kinase suppressor of Ras
GPCR: G protein-coupled receptor
UPS: ubiquitin–proteasome system
EGF: epidermal growth factor
FSK: Forskolin
RTK: receptor tyrosine kinase
bFGF: basic fibroblast growth factor
ESC: embryonic stem cell
EpiSC: epiblast-like stem cell
