# Supplementary material for: praja2 regulates KSR1 stability and mitogenic signaling
Source: Cell Death Dis. 2016 May 19;7(5):e2230–. doi: 10.1038/cddis.2016.109 (PMC4917648; doi:10.1038/cddis.2016.109)
Supplement: Supplementary Information [file cddis2016109x1.pdf]

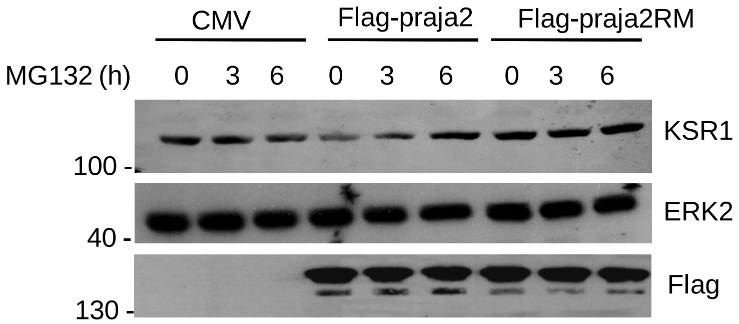

Supplementary figure S1. praja2 degrades KSR1 through the proteasome. Immunoblot of lysates from cells transiently transfected with Flag-praja2 vector (either wild type or ring mutant). Where indicated, the cells were treated with MG132 (10μM) and harvested at the indicated time points.

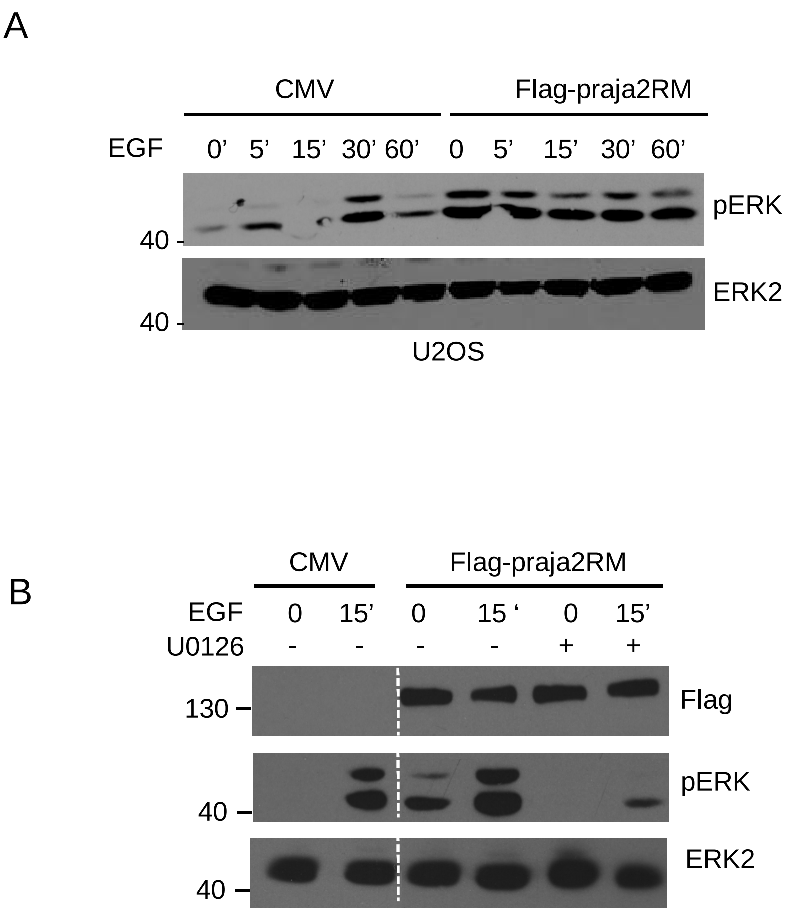

Supplementary Figure S2. praja2 regulates ERK phosphorylation by EGF. Osteosarcoma cells (U2OS) (**A**) or HEK 293 cells (**B**) were transiently transfected with empty vector (CMV) or flag-praja2rm (RM) vector and treated with EGF (100ng/ml). HEK293 are pretreated for 15 minutes with U0126 (5µm) (**B**). Lysates were immunoblotted with the indicated antibodies.

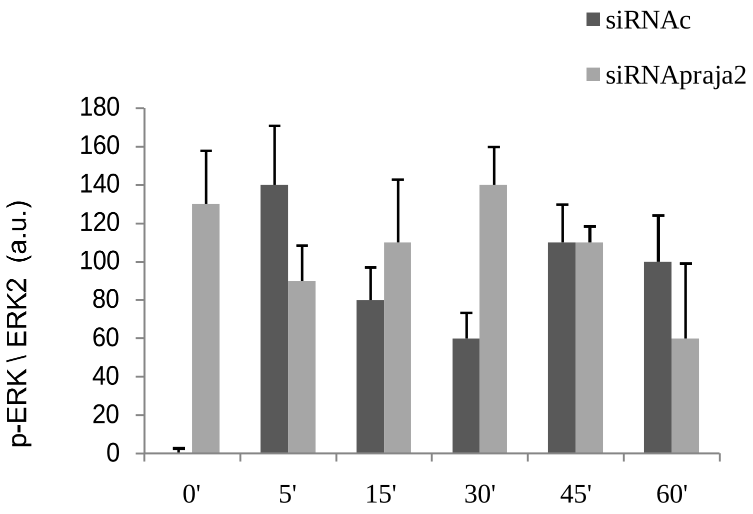

Suplementary figure S3. Quantitative analisys of esperiment showed in 4C .

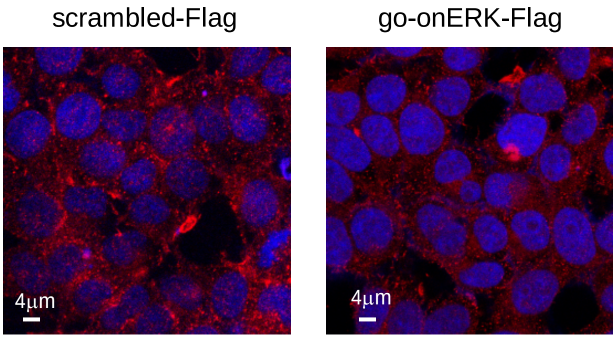

Suplementary figure S4. Entry of go-onERK-Flag into the cells. HEK293 cells, treated for eight hours with scrambled-Flag or go-onERK-Flag peptides, were immunostained with monoclonal anti-Flag antibody. Nuclei were stained with DRAQ5. Images were collected and analyzed by confocal microscopy.

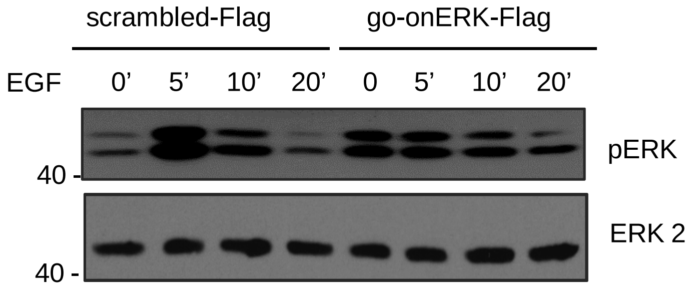

LNCAP

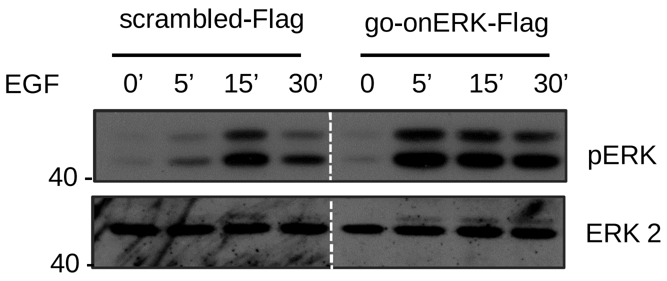

U87

Suplementary figure S5. LNCAP and U87 cells were pretreated (8 hours) with the synthetic peptides(1 µM) (scrambled-Flag and go-onERK-Flag-Flag) and stimulated with EGF. Lysates were immunoblotted for P-ERK and ERK

Kinase domain of KSR2 is in PDB (2Y4I)
Sequence alignment with KSR1 shows very high conservation
Identified Peptide 862-DLQERPSFSL-872 in KSR1 is analogous to 913-EQEERPTFTK-923

```
SP|Q8IVT5|KSR1_HUMAN MDRAALRAAAMGEKKEGGGGGDAAAAEGGAGAAASRALQQCGQLQKLIDISIGSLRGLRT 60
SP|Q6VAB6|KSR2_HUMAN ----------MDE-------ENMTKSEEQQPLSLQKALQQCELVQNMIDLSISNLEGLRT 43
                               * *        : : :*     : .:*****  :*::**:**..*.****

SP|Q8IVT5|KSR1_HUMAN KCAVSNDLTQQEIRTLEAKLVRYICKQRQCKLSVAPGERTPELNSYPRFSDWLYTFNVRP 120
SP|Q6VAB6|KSR2_HUMAN KCATSNDLTQKEIRTLESKLVKYFSRQLSCKKKVALQERNAELDGFPQLRHWFRIVDVRK 103
                     ***.******:******:***:*:.:* .** .**  **. **:.:*:: .*:  .:**

SP|Q8IVT5|KSR1_HUMAN EVVQEI-PRDLTLDALLEMNEAKVKETLRRCGASCDECGRLQYALTCLRKVTGLGGEHKE 179
SP|Q6VAB6|KSR2_HUMAN EVLEEISPGQLSLEDLLEMTDEQVCETVEKYGANREECARLNASLSCLRNVHMSGGNLSK 163
                     **::** * :*:*: ****.: :* **:.: **. :**.**: :*:***:*   **: .:

SP|Q8IVT5|KSR1_HUMAN DSSWSSLDARRESGSCPSTDTLSAASLPWPPGSSQLG-RA-----------GNSAQGP-- 225
SP|Q6VAB6|KSR2_HUMAN -QDWTIQWPTTETGKENN-PVCPPEPTPWIRTHLSQSPRVPSKCVQHYCHTSPTPGAPVY 221
                      ..*:      *:*.  .  .      **     . . *.           . :  .*

SP|Q8IVT5|KSR1_HUMAN ---RSISVSALPASDSPTPSFSEGLSD-TCIPLEA-SGRLT--PRALHSFITPPTTPQLR 278
SP|Q6VAB6|KSR2_HUMAN THVDRLTVDAYPGLCPP-PPLESGHRSLPPSPRQRHAVRTPPRTPNIVTTVTPPGTPPMR 280
                          ::*.* *.   * * :..*  .    * :  : *       : : :*** ** :*

SP|Q8IVT5|KSR1_HUMAN RHTKLKPPRTPPPPSRKVFQLLPSFPTLTRSKSHESQLGNRIDDVSSMR----------- 327
SP|Q6VAB6|KSR2_HUMAN KKNKLKPPGTPPPSSRKLIHLIPGFTALHRSKSHEFQLGHRVDEAHTPKAKKKSKPLNLK 340
                     ::.***** **** ***:::*:*.* :* ****** ***.*:*:. : :

SP|Q8IVT5|KSR1_HUMAN ----------------------------------------------------FDLSHGSP 335
SP|Q6VAB6|KSR2_HUMAN IHSSVGSCENIPSQQRSPLLSERSLRSFFVCHAPFLPSTPPVHTEANFSANTLSVPRWSP 400
                                                                         :.: : **

SP|Q8IVT5|KSR1_HUMAN QMVRRDIGLSVTERFSTKSWLSQVCHVCQKSMIFGVKCKHCRLKCHNKCTKEAPACRISF 395
SP|Q6VAB6|KSR2_HUMAN QIPRRDLGNSIKHRFSTKYWMSQTCTVCGKGMLFGLKCKNCKLKCHNKCTKEAPPCHLLI 460
                     *: ***:* *:..***** *:**.* ** *.*:**:***.*:************ *:: :

SP|Q8IVT5|KSR1_HUMAN LP---LTRLRRTESVPSDINNPVDRAAEPHFG----TLPK-ALTKKEHPPAMNHLDSSSN 447
SP|Q6VAB6|KSR2_HUMAN IHRGDPARLVRTESVPCDINNPLRKPPRYSDLHISQTLPKTNKINKDHIPVPYQPDSSSN 520
                     :     :** ******.*****: :  .        ****    :*:* *.  : *****

SP|Q8IVT5|KSR1_HUMAN PSSTTSSTPSSPAPFPTSSNPSSATTPPNPSPGQRDSRFNFPAAYFIHHRQQFIFPVPSA 507
SP|Q6VAB6|KSR2_HUMAN PSSTTSSTPSSPAPPLPPSATPPSPLHPSPQCTRQQKNFNLPASHYYKYKQQFIFPDVVP 580
                     **************    *    :   *.*.  :::..**:**::: :::******

SP|Q8IVT5|KSR1_HUMAN GHCWKCLLIAESLKENAFNISAFAHAAPLPEAADGTRLDDQPKADVLEAHEAEAEEPEAG 567
SP|Q6VAB6|KSR2_HUMAN VPET--------PTRAPQVILHPVTSNPILEGNPLLQIEVEPTS--------EN---EEV 621
                                  ..    *   . : *: *.   ::: :*.:         *    *

SP|Q8IVT5|KSR1_HUMAN KSEAEDDEDEVDDLPSSRRPWRGPISRKASQTSVYLQEWDIPFEQVELGEPIGQGRWGRV 627
SP|Q6VAB6|KSR2_HUMAN HDEAEESEDDFEEMNLSL-LSARSFPRKASQTSIFLQEWDIPFEQLEIGELIGKGRFGQV 680
                     :.***:.**:.:::  *       : *******::**********:*:** **:**:*:*

SP|Q8IVT5|KSR1_HUMAN HRGRWHGEVAIRLLEMDGHNQDHLKLFKKEVMNYRQTRHENVVLFMGACMNPPHLAIITS 687
SP|Q6VAB6|KSR2_HUMAN YHGRWHGEVAIRLIDIERDNEDQLKAFKREVMAYRQTRHENVVLFMGACMSPPHLAIITS 740
                     ::***********:::: .*:*:** **:*** *****************.*********

SP|Q8IVT5|KSR1_HUMAN FCKGRTLHSFVRDPKTSLDINKTRQIAQEIIKGMGYLHAKGIVHKDLKSKNVFYDNGKVV 747
SP|Q6VAB6|KSR2_HUMAN LCKGRTLYSVVRDAKIVLDVNKTRQIAQEIVKGMGYLHAKGILHKDLKSKNVFYDNGKVV 800
                     :******:*.*** *  **:**********:***********:*****************

SP|Q8IVT5|KSR1_HUMAN ITDFGLFGISGVVREGRRENQLKLSHDWLCYLAPEIVREMTPGKDEDQLPFSKAADVYAF 807
SP|Q6VAB6|KSR2_HUMAN ITDFGLFSISGVLQAGRREDKLRIQNGWLCHLAPEIIRQLSPDTEEDKLPFSKHSDVFAL 860
                     *******.****:: ****::*::.. ***:*****:*:::* .:**:***** :**:*:

SP|Q8IVT5|KSR1_HUMAN GTVWYELQARDWPLKNQAAEASIWQIGSGEGMKRVLTSVSLGKEVSEILSACWAFDLQER 867
SP|Q6VAB6|KSR2_HUMAN GTIWYELHAREWPFKTQPAEAIIWQMGT--GMKPNLSQIGMGKEISDILLFCWAFEQEER 918
                     **:****:**:**:*.* *** ***:*:  ***  *:.:.:***:*:**  ****: :**

SP|Q8IVT5|KSR1_HUMAN PSFSLLMDMLEKLPKLNRRLSHPGHFWKSADINSSKVVPRFERFGLGVLESSNPKM 923
SP|Q6VAB6|KSR2_HUMAN PTFTKLMDMLEKLP                  ------------------------ 950
                     *:*: ********** **************::
```

Suplementary figure S6. alignament of human KSR1 (KSR1_HUMAN, Q8IVT5) with KSR2 sequence (KSR2_HUMAN, Q6VAB6)
